# Observations on the Profession of Apothecary

**Published:** 1813-07

**Authors:** 


### *To the Editors of the Medical and Physical Journal.*

GENTLEMEN,

THE Preamble to the "Act for exempting Apothecaries from certain Offices, &c." Anno 6-7 Will. III. runs thus: "Whereas the art of the Apothecary is of great and general use and benefit, by reason of their constant and necessary *assistance* to his Majesty's subjects, *which should oblige them solely to attend the duties of their profession;* yet, by reason that they are compelled to serve several parish, ward, and leet offices, in the places where they live, and are frequently summoned to serve on juries and inquests, which take up great part of their time, they cannot perform *the trusts reposed in them* as they ought, nor *attend the sick with such diligence* as they ought, &c."

I would beg to ask your learned correspondent, Censorinus, whether it is a just and legitimate deduction from the manner in which the words "assistance," "attend," "duties," "profession," are used in the above extract, to conclude that the Apothecary's attendance upon the sick was to be somewhat like that of the nurse, or whether they do not imply that he was to prescribe for them? If your correspondent's opinion respecting the Apothecaries be correct, they are, I apprehend, one and all liable to a penalty of 5*l.* per month for practising; but if they are by the above act allowed to exercise their "profession," then have they as much a right to prescribe for patients as the physicians have.

The disputes at present subsisting between physicians and apothecaries, have caused me to turn over some old tracts upon the same subject: from one of these I find that the apothecaries had very little employment as prescribers of medicine till the time of the great plague in 1666, when the *physicians* taking the alarm, *honestly* and *disinterestedly* ran away, but the *apothecaries* kept their proper posts,* and as far as they were able assisted the sufferings of their fellow-creatures; after which they rose into estimation, and from that time to this have either legally or illegally practised.

In another pamphlet I found the following paragraph, which I have been induced to extract for the amusement of your readers: "After all it must be owned that there is something very peculiar in the œconomy of an Apothecary, and his brain is very much influenced by the weather. On a calm fair day he is very ignorant and stupid; but in the night, and when it rains, he is a man of common sense, and

* See Merrett's Short View of Frauds and Abuses, &c. &c. 1669.

a competent a competent judge of diseases. This may be proved from the general acknowledgment of physicians themselves, who do not pretend to prescribe to a patient before they know his disease; and he who does not understand a distemper himself, can never give an exact relation of it to another. Now, if a physician's skill be required in the night, or on a rainy day, and he sends for the apothecary, orders him to visit the patient and bring him an account of the case, and then prescribes for the sick person remedies without seeing him himself, this is an acknowledgment of the apothecary's judgment; for it would be barbarous to say of any physician, that he preferred his own ease to his patient's safety."—*Pharmacopolæ justificati, or Apothecaries vindicated from the Imputation of Ignorance.* 1756.

*June* 10, 1813. A. A. Y.

